# Repair of Complex Esophageal Atresia with Tracheobronchial Remnant using Special Magnets

**DOI:** 10.1055/s-0044-1779042

**Published:** 2024-02-02

**Authors:** Charlotte Reich, Elena Weigl, Anne-Sophie Holler, William Lee, Michael Harrison, Oliver J. Muensterer

**Affiliations:** 1 Department of Pediatric Surgery, Munich University Hospital Dr von Hauner Children's Hospital, Munchen, Bavaria, Germany; 2 Department of Pediatric Surgery, University of California San Francisco, San Francisco, California, United States

**Keywords:** esophageal atresia, tracheobronchial remnant, magnet, anastomosis, thoracotomy

## Abstract

Esophageal atresia (EA) repair can be complicated by associated malformations such as a tracheobronchial remnant in the distal esophagus. We describe our experience with a patient found to have long-gap EA with a distal cartilaginous ring who was managed using a combination of esophageal lengthening and magnetic compression anastomosis. A 5-month-old girl was referred to us from an outside hospital with type C EA including a very high upper pouch. She had undergone a prior thoracotomy with fistula ligation during which a clip was placed on the lower esophagus, leaving a 2-cm diverticulum on the trachea and a short lower esophageal pouch. Upon endoscopic evaluation at our center, we found a tracheobronchial remnant in the lower esophagus between the clip and the carina. An open thoracotomy was performed to approximate the esophageal pouches and a magnet anchor (Connect EA, Myka Laboratories, San Francisco, California, United States) was placed retrograde through the distal esophageal cartilaginous ring into the lower pouch. On postoperative day 8, after adequate growth and decreased pouch tension, a second magnetic anchor was placed endoscopically to the upper pouch to mate with the previously placed lower pouch anchor. The anastomosis formed within 14 days. Due to the tracheobronchial remnant, the device did not pass distally and was removed endoscopically. On postoperative day 8, balloon dilation of the anastomosis and tracheobronchial remnant was performed. Subsequently, the patient required a total of 6 dilations in an 18-month follow-up. This case report illustrates the utility of using magnets to create an esophageal anastomosis in complex cases of EA with concomitant esophageal malformations. The parents of the patient gave their written consent to publish this technical report.

## Introduction

Repair of complex esophageal atresia remains a challenge, and since the spectrum of associated malformations is vast, it is important for the pediatric surgeon to expect the unexpected. While short-term goals include the separation of the aerodigestive tracts distal to the glottis, as well as establishment of esophageal continuity, this should be achieved in the least invasive manner possible. Repeated thoracotomies carry the risk of lung injury, rib fusion, scoliosis, and other musculoskeletal disorders that can compromise the long-term goals of functional normalcy. 1 2

The Connect-EA (Myka Laboratories, San Francisco, California, United States) device is a pair of uniquely engineered magnetic anchors designed to create an esophageal anastomosis within 8 to 10 days after placement. They have been tested extensively in animal studies 3 4 before using them in select clinical cases. 5 6 7

This report discusses the technical management of a patient with esophageal atresia and distal tracheoesophageal fistula (gross type C) that was found to have a distal esophageal tracheobronchial remnant after previous ligation of the lower esophagus. This approach utilized the Connect-EA magnetic device to create an esophageal anastomosis and avoided repeated thoracic interventions.

The parents of the patient gave their written consent to publish this technical report.

## Case Report

**Fig. 1:**
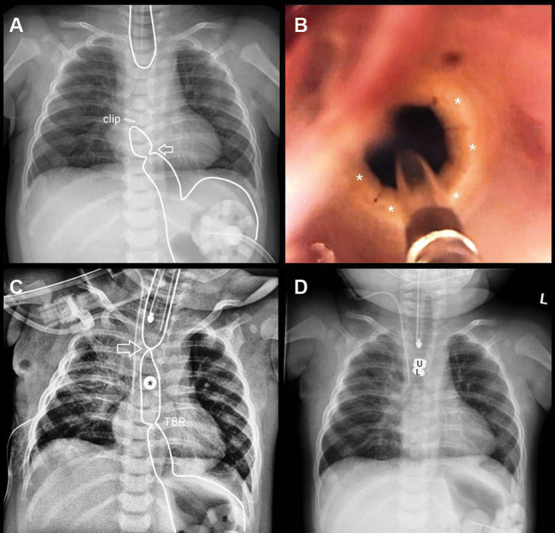
( **A** ) Contour of the upper and lower esophageal pouches (white lines) at the time of broncho-esophagogastroscopy at our center showing the clip ∼2 cm distal to the carina (clip) and the waist at the location of the tracheobronchial remnant (arrow). ( **B** ) Endoscopic view of the first attempt at balloon dilatation of the tracheobronchial remnant. The cartilaginous ring is appreciable through the inflated balloon indicated by the white line (asterisks). ( **C** ) Postoperative chest radiograph after approximation and placement of one Connect-EA magnetic anchor into the lower esophagus above the tracheobronchial remnant (TBR). ( **D** ) Two stacked, endoscopically introduced magnetic anchors in the upper esophageal pouch (U) mated with the previously placed magnetic anchor in the lower esophageal pouch (L).

A 5-month-old girl born at an outside hospital in a low-resource country was referred to our center for further management of a complex esophageal atresia. She had undergone a prior thoracoscopic exploration, during which the upper pouch was found to be located high in the neck. Efforts at mobilization were abandoned, the procedure was converted to an open thoracotomy, the lower esophagus was ligated with a metal clip, and a gastrostomy was created. Upon endoscopic exploration at our center, we identified a 2-cm diverticulum from the posterior wall of the trachea, as well as a short lower esophageal pouch that was interrupted by a tracheobronchial remnant in the form of a tight cartilaginous ring ( Fig. 1A ). To visualize the entire lower esophagus, a wire was placed under fluoroscopy and an unsuccessful attempt was made to dilate the tracheobronchial remnant with balloon dilation over the guidewire ( Fig. 1B ).

The plan was made to perform an initial approximation of the esophageal ends for later magnetic compression anastomosis. The challenge was to bring the lower magnet into the upper portion of the lower esophageal pouch, cranial to the tracheobronchial remnant. This was not possible thoracoscopically, and the procedure was converted to a posterolateral thoracotomy. After mobilizing and suture approximating the esophageal ends under tension, one of the Connect-EA magnetic anchors was introduced endoscopically via the gastrostomy into the lower esophagus. Using digital manipulation and an upward milking motion, the magnet was translocated into the upper portion of the lower esophagus beyond the cartilaginous ring of the tracheobronchial remnant. While the magnetic anchor was in place, the patient exhibited noisy breathing resembling tracheomalacia. The patient was given 8 days with the magnet in place for the approximation to mature and the tension to subside ( Fig. 1C ).

**Fig. 2:**
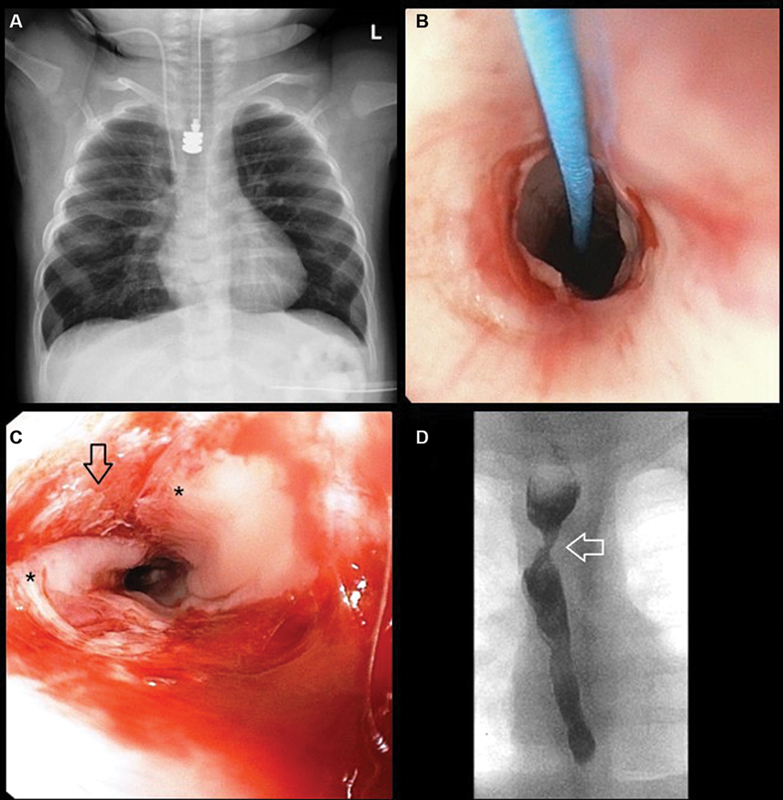
( **A** ) After 10 days, all three magnetic anchors were completely juxtaposed and saliva was found draining from the gastrostomy, indicating that the anastomosis had formed. However, the magnets did not pass distally spontaneously. ( **B** ) Eight days after device removal, endoscopy demonstrated a widely patent esophageal anastomosis. ( **C** ) The tracheobronchial remnant was slowly and progressively dilated until it ruptured. The image shows the separated ends of the cartilaginous ring (*) and the resulting mucosal defect (arrow). Care must be taken not to overdilate and thereby cause full-thickness disruption of the esophagus. ( **D** ) An esophagram performed 1 month after magnet removal showed a patent esophagus with mild degree of narrowing at the site of the anastomosis (arrow).

After 8 days, peroral endoscopic magnetic anchor placement was performed. Initially, the thickness of the esophageal tissue precluded a single upper anchor from mating sufficiently, so an additional Connect-EA magnetic anchor was stacked on top of the upper anchor to increase the magnetic force and facilitate a firm and secure attachment ( Fig. 1D ). After 14 days, saliva started to drain from the gastrostomy, indicating patent anastomosis formation ( Fig. 2A ). However, due to the tracheobronchial remnant, the magnets did not pass distally, and peroral endoscopic removal was required.

Eight days after device removal, the anastomosis was widely patent on endoscopy ( Fig. 2B ) and the tracheobronchial remnant was slowly dilated with increasing diameter until the cartilaginous ring ruptured at 12-mm diameter ( Fig. 2C ). This procedure was tolerated well and the patient started oral feeds on postoperative day 2. An esophagram performed 1 month after device removal demonstrated a patent esophagus with a waist at the site of anastomosis ( Fig. 2D ). At 18-month follow-up, the patient has required a total of six balloon dilations with no additional dilations anticipated. The patient is currently on full oral feeds.

## Discussion

This case highlights how the principles of esophageal magnetic compression anastomosis can be applied in complex cases of esophageal atresia to decrease invasiveness and obtain a satisfactory anatomic and functional outcome. 6 In our patient, primary anastomosis during attempted conventional repair was precluded by high tension associated with a long (five vertebral body) gap. The tracheobronchial remnant further complicated the issue, because resection and reanastomosis at the time of approximation would have carried a high risk of disruption and leak due to the high tension as well. Placing the first magnetic anchor above and beyond the cartilaginous ring during the approximation procedure allowed for later endoscopic introduction of magnetic anchors in the upper pouch with subsequent mating and anastomosis.

Unfortunately, one of the all-too-common pitfalls in patients referred to our center from the outside is the ligation of the lower esophagus inappropriately distal, leaving a long diverticulum off the trachea and causing an “iatrogenic long-gap esophageal atresia.” This entity can be avoided by spending extra effort and time during the primary operation to dissect the lower esophagus all the way to the origin of the tracheoesophageal fistula, and ligating it as high up as possible.

The concept of delayed primary anastomosis to treat long-gap esophageal atresia includes waiting for the esophagus to grow proportionally with time. Interestingly in this case, spontaneous upper pouch elongation did not occur. In our experience, failure of spontaneous distal elongation of the upper pouch can be associated with several factors. First, an aberrant subclavian vessel may represent an anatomic barrier for spontaneous distal growth. 8 9 However, the vasculature was normal in our patient. Second, scars and adhesions from prior surgical dissection can preclude the natural elongation of the upper pouch. Since the patient had undergone a previous thoracoscopic attempt at anastomosis, scarring resulting from the first procedure was most likely the underlying reason for the lack of spontaneous distal expansion. This notion is also supported by the fact that dense adhesions were appreciated upon mobilizing the upper pouch during the approximation procedure at our center.

An interesting observation during treatment of this case was the transient development of tracheomalacia. Tracheomalacia results from a combination of intrinsic and extrinsic factors. Patients with esophageal atresia often have an intrinsic predisposition to tracheomalacia due to a soft and underdeveloped tracheal structure. 10 However, in this case, the findings most likely occurred due to mass effect from the magnetic anchors in the esophageal segments displacing the back wall of the trachea anteriorly. Our hypothesis is supported by the fact that the noisy breathing immediately subsided once the magnets were removed. Although the symptoms were mild and did not require any form of therapy, the possibility of transient tracheomalacia should be discussed as a potential risk with the parents of candidates for esophageal magnetic compression anastomosis during the informed consent process.

A limitation of our report is that the cartilaginous ring was not proven by histopathology. Although we did not perform a resection of the tissue, the endoscopic appearance certainly ( Fig. 1B ) was highly typical of a cartilaginous ring in the context of a tracheobronchial remnant. However, an unusual type of segmental fibromuscular hypertrophy (FMS) or other type of congenital stenosis cannot be completely ruled out.

Usually after 8 to 10 days postplacement, the magnetic anchors detach from the future anastomotic site, pass distally through the gastrointestinal tract, and are eliminated via stool. However, in this case, the presence of a tracheobronchial remnant precluded the distal passage of the magnets, requiring peroral endoscopic retrieval. This intervention gave us the opportunity to inspect the newly formed anastomosis.

Had our patient not been treated with magnetic compression anastomosis in the described fashion, she would have most certainly required at least one additional thoracotomy for the esophageal anastomosis, and possibly another surgical intervention for the tracheobronchial remnant. 11 Performing a primary anastomosis at the time of thoracotomy at our center was precluded due to the extremely high amount of tension between the pouches. An additional thoracotomy would have potentially placed her at risk for well-known sequelae resulting from repeat thoracotomy, such as rib fusion, scoliosis, and nerve injury leading to scapula alata. 1 2 12 With the proposed magnetic compression anastomosis approach, an expected avoidance of multiple highly invasive procedures and the potential to minimize harm to the patient served as the basis for compassionate care justification, according to the principles of our university's ethical review board.

## Conclusion

This technical report details the management of a complex form of long-gap esophageal atresia with a lower tracheobronchial remnant using the Connect-EA device for esophageal magnetic compression anastomosis, expanding its utility and indications. The general principle of the method is based on approximating the esophageal ends under tension, waiting for the tension to subside, and then delivering the magnetic device to gradually form a secure anastomosis. While the Connect-EA device has been deployed in a variety of clinical scenarios so far, the concept of placing the magnets in a metachronous fashion is novel and a promising approach for these types of complex cases.
